# Mechanobiological evaluation of solid and multiple porous humeral stem architectures in reverse shoulder arthroplasty based on design and materials: a finite element study

**DOI:** 10.3389/fbioe.2025.1675726

**Published:** 2026-02-02

**Authors:** Pearline Beulah John, Sharmila Nageswaran

**Affiliations:** Department of Sensor and Biomedical Technology, School of Electronics Engineering, Vellore Institute of Technology, Vellore, India

**Keywords:** finite element analysis, humeral stem, porous stems in RSA, stress shielding in RSA, Ti-6Al-4V, trabecular architecture

## Abstract

**Introduction:**

Stress shielding is a major cause of radiological changes in the humeral component, which is commonly evident in cementless stems of reverse shoulder implants. The bulkiness of the humeral stem results in less load being transmitted to the bone, curtailing bone remodeling. Designing an implant with adequate strength and a suitable material that matches the mechanical properties of bone can help prevent the implant migration or loosening, thereby lowering bone resorption.

**Methods:**

Humeral stems with no porosity and varied porosities, such as circular, elliptical, and trabecular architecture, were designed using 316L stainless steel and titanium alloy (Ti6Al4V). Finite element analysis (FEA) was conducted on eight bone–implant assemblies under four loading conditions for cortical and trabecular bones. Weighted mean of von Mises stress and mechanobiology associated with the strain energy densities were studied. This serves as a precursor in predicting the effect of stress shielding.

**Results:**

The titanium implant with trabecular architecture was mechanically close to the intact bone compared to the other varied porosity designs. It also had better load-bearing capacity than the solid stems.

**Discussion:**

These investigations help understand the load-bearing capacity of reverse shoulder humeral stems and ascertain the importance of combining the design and material in enhancing implant stability and longevity.

## 1 Introduction

Shoulder replacement procedures have increased in the United States over the past decade (Dillon et al., 2017; Wagner et al., 2020; Kim et al., 2011) owing to the implementation of reverse total shoulder arthroplasty (RTSA) after being approved by the Food and Drug Administration (FDA) in 2003 (Dillon et al., 2017; Wagner et al., 2020; Kim et al., 2011). Internationally, an annual increase was observed in the recording of several shoulder registries (Lübbeke et al., 2017). It has been reported that the rate of incidence for shoulder arthroplasty (total and reverse) procedures has increased to 12% and 32%, respectively, during the years 2005–2013 (Wagner et al., 2020). Since the increase in shoulder replacement procedures is slightly higher than or proportionate to that of hip and knee arthroplasty surgeries, the need for shoulder joint replacement can surpass them in the years to come (Day et al., 2010).

RTSA was first proposed by Paul Grammont in 1985 for the treatment of massive rotator cuff injuries since the anatomic prosthesis used in total shoulder arthroplasty (TSA) was unable to restore the functionality of the joint (Boileau et al., 2005). This design encountered failure as they were cemented, and only two components were involved. Subsequently, this has given rise to Delta III, which is now in use and manufactured in the market (Ackland et al., 2015). Although successive designs focused on improving glenoid attachment and reducing scapular notching, one of the most significant challenges observed was stress shielding in the humeral stem (Mazaleyrat et al., 2021). RTSA is a promising shoulder replacement procedure for cases of severe rotator cuff muscle injuries, osteoarthritis, and complicated shoulder pathological conditions (Takayama and Ito, 2024).

Since the introduction of the modern reverse shoulder arthroplasty by Grammont, humeral components have undergone substantial modifications. Traditional humeral stems that measured between 100 and 150 mm, were dependent on canal filling. Later, this caused proximal osteolysis, cortical thinning, and tuberosity resorption due to the effects of stress shielding. Modern designs adapted short stems (<100 mm) to reduce intramedullary engagement, reduce stress shielding, and improve stem removal during revision surgery (Lehman et al., 2024). Initial versions of RTSA stems used cemented forms. However, studies suggest that cementless stems are used nowadays due to the consequences that arise during their revision surgeries. In addition, thrombosis and the long duration of the operative procedures have led to use of cementless stems; cementless stems lead to promising bone preservation (Takayama and Ito, 2024; Nakazawa et al., 2023).

In addition to stem length, the latest findings have emphasized the significance of diaphyseal filling and metaphyseal filling ratios in the prediction of adaptive changes in the proximal bone. It is reported that stress shielding and cortical thinning increased with high filling ratios (Lehman et al., 2024), resulting in metaphyseal-focused fixation techniques in modern humeral stem designs.

Recent developments in additive manufacturing (AM) technology have contributed to the development of complex joint prosthesis, products that mimic the properties of human bone, scaffolds, and medical devices that are cumbersome to produce using traditional manufacturing methods (Zadpoor, 2017). The main significance of the AM technology is that it provides precise control over the implant’s geometry and internal architecture to meet the required mechanical properties, thereby reducing cost and material usage (Wieding et al., 2014). Studies suggest that porous implants manufactured using AM technology can impart optimal stiffness and overall stability to the bone and implant (Kawai et al., 2017; Tarlochan et al., 2018; Li et al., 2019). Hence, additive manufacturing has rendered a viable solution to the problem caused by the usage of porous stems, which can potentially alleviate the outcomes of reduced mechanical stimulus, thereby ameliorating the longevity of humeral stems (Soltanmohammadi et al., 2022).

Stress shielding is one of the predominant factors affecting implant longevity and is a significant contributor to radiological changes in the humeral component, which are commonly evident in cementless humeral stems (Mazaleyrat et al., 2021). Wolff’s law states that the bone of an individual constantly remodels to the stress it is subjected to (Nagels et al., 2003). However, prosthesis implantation results in bone density deprivation as excess load is transferred to the implant rather than the bone. The result of large loads being transferred to the implant causes a discrepancy in the rigidity between the implant and native bone, leading to osteolysis, causing implant migration, thereby increasing the probability of revision surgeries (Hitchon et al., 2024).

Stress shielding was found to be more prevalent in patients after cementless stem implantation (Vasiliadis et al., 2024). It can be determined by ascertaining variations in the density of the proximal humerus (Melis et al., 2011). Major causes of failure of the reverse humeral stems arise in the upper region (proximal) of the humerus, where osteolysis of the bone is observed (Ascione et al., 2018; Boileau, 2016; Boileau et al., 2013; Grey et al., 2018), causing stress shielding.

Recently, a continued surveillance of RTSA with stems larger than 100 mm revealed that it was associated with fractures in the humerus and prosthetic loosening of the humeral stem, leading to ramifications in the functional outcomes. Hence, all these factors contribute to the need for revision surgeries (Ascione et al., 2018; Grey et al., 2018). Humeral stem design plays a significant role in transferring load to the bone and thus bone remodeling. Long stems can result in stress shielding and osteolysis (Vasiliadis et al., 2024). Moreover, it has been demonstrated that short stems generate bone stress that resembles that of the complete humerus bone (Razfar et al., 2016).

Preliminary clinical results of short humeral stems have been favorable (Tross et al., 2020). However, radiolucent lines, osteopenia, aseptic stem loosening, cortical thinning, risk of misalignment, and loosening due to improper bone ingrowth are still prevalent (Tross et al., 2020; Giuseffi et al., 2014). In a 2-year follow-up related to use of short stems in 77 RSAs, stress shielding was evident in 35% of the humeral stems (Raiss et al., 2019). Although short stems overcome the limitations of long humeral stems, their potential disadvantage arises due to the lack of long-term clinical studies associated with them (Giuseffi et al., 2014).

Hollow and porous hip implants decrease the stiffness of implants and enhance their functionality (Liverani et al., 2021; Wang et al., 2020; Arabnejad et al., 2017; Alkhatib et al., 2019; Sivarasu et al., 2011). Hollow humeral stems (Soltanmohammadi et al., 2022) and porous humeral stems (Hitchon et al., 2024) for TSA have been investigated. Nevertheless, despite the advancements in stem length, metaphyseal fixation, and canal filling, AM-generated porous humeral stems for RSA have not been studied extensively. In particular, there are only a few supporting studies on how various porous architectures determine proximal stress transfer and strain energy distribution zone-wise. Furthermore, the effects of lattice geometry of the humeral stems for RSA and comparative studies focusing on the porosity and material types have not been analyzed.

The fundamental purpose of this work is to evaluate the effect of mechanical stimulus on the humeral implant (for RTSA) and bone based on porosity and materials using finite element analysis. This approach can help evaluate the stress-shielding signal and determine the impact of the implantation of solid and porous stems on the bone.

## 2 Materials and methods

### 2.1 3D modeling of humerus

**FIGURE 1:**
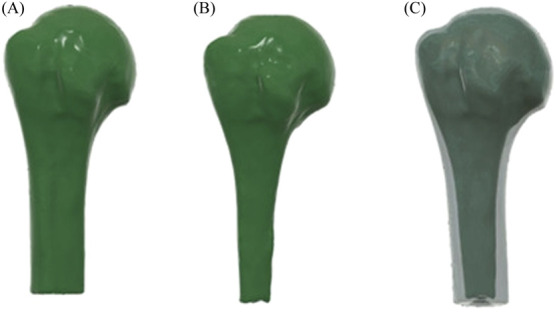
3D model humerus: **(A)** cortical bone; **(B)** trabecular bone; **(C)** assembled cortical and trabecular bone.

The humerus model was created from the CT scan images of the shoulder, obtained from Kaggle, a repository that hosts publicly available datasets (https://www.kaggle.com/datasets/syxlicheng/automatically-transform-ct-datasets-into-drrs). The images were obtained in DICOM format; cortical and trabecular bones were segmented using 3D Slicer. The CT DICOM dataset consisted of 158 slices with a matrix size of 512 × 512 pixels, a pixel size of 0.5977 mm, slice thickness of 1.0 mm, and slice increment of 1.0 mm. The cortical bone was segmented in the threshold range of 700–2000 HU, and trabecular bone was segmented in the threshold range of 150–300 HU (Zhao et al., 2018) and reconstructed as 3D models, imported to Fusion 360, and assembled as shown in Figure 1.

### 2.2 3D modeling of reverse shoulder humeral stems

The humeral stem is replaced after a considerable portion of the bone from the intramedullary canal is reamed. Humeral stems were designed using the reverse engineering approach. The dimensions were taken from published studies and from standard measurements available in commercial implants. A solid stem was first designed, and later, different porous geometries, such as circular, elliptical, and trabecular-like lattice architectures, were introduced in the proximal metaphyseal region to develop other models. These designs were developed by maintaining the overall stem shape and dimensions across all models.

**FIGURE 2:**
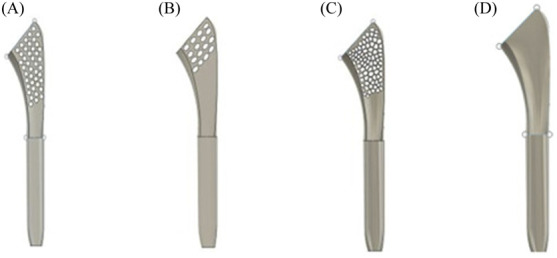
Design of the humeral stem with varying patterns: **(A)** circular pattern; **(B)** elliptical; **(C)** trabecular architecture; **(D)** solid stem.

**FIGURE 3:**
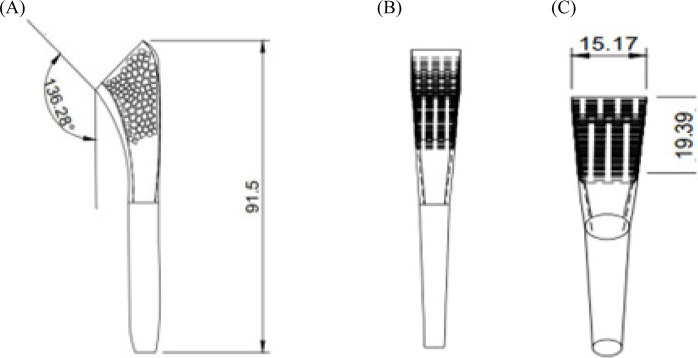
Trabecular architecture in different planes: **(A)** X–Y; **(B)** X–Z; **(C)** Y–Z plane.

Four humeral stems of length 90 mm were developed using Fusion 360 as using short stems of length 90 mm may provide increased resistance to bone migration (Bents et al., 2022). A neck shaft angle of 135° was set as previous studies have indicated that this angle decreases the prevalence of scapular bone erosion and enhances joint mobility (Sheth and Saltzman, 2019; Neyton et al., 2023; Holsters et al., 2021). One of the stems was developed as a solid model, and three others were developed with varying porosities at the proximal humerus. Figure 2 represents four different types of humeral stems with various patterns, such as solid, circular, elliptical, and a pattern similar to trabecular architecture (so that it can mimic the intact bone, thereby augmenting osteointegration) of the bone. The trabecular architecture was designed using the Voronoi sketch generator in Fusion 360. Figure 3 shows the diagrams of the humeral stem with trabecular architecture on all planes.

### 2.3 Finite element model: assembly and meshing

**FIGURE 4:**
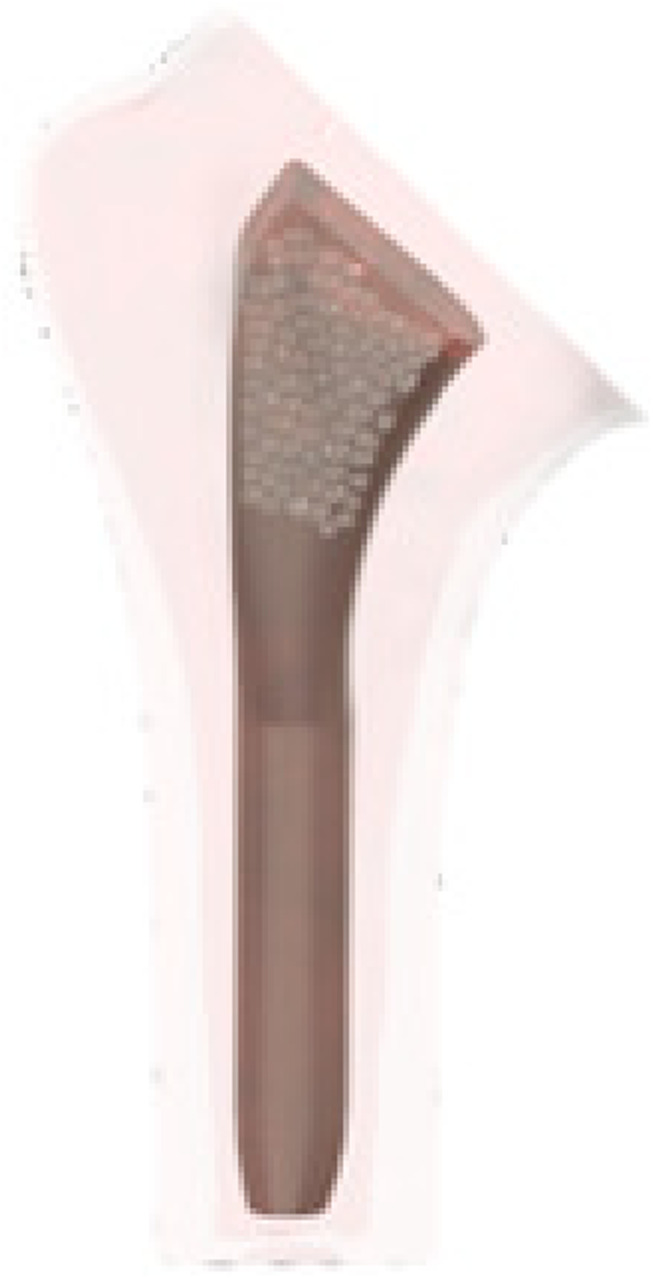
Assembly of the bone and implant with trabecular architecture.

The head of the humerus was virtually osteotomized, and the titanium humeral stem with the trabecular pattern was assembled with cortical–trabecular bone, as shown in Figure 4.

**FIGURE 5:**
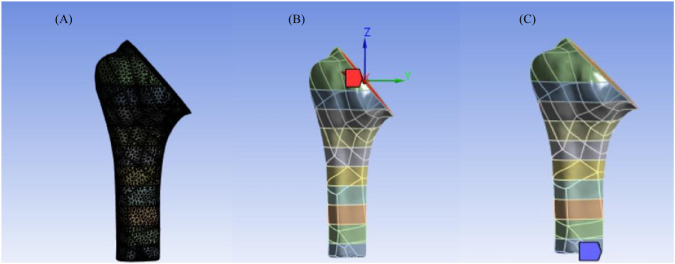
Finite element model: **(A)** mesh; **(B)** loading; **(C)** boundary conditions.

**FIGURE 6:**
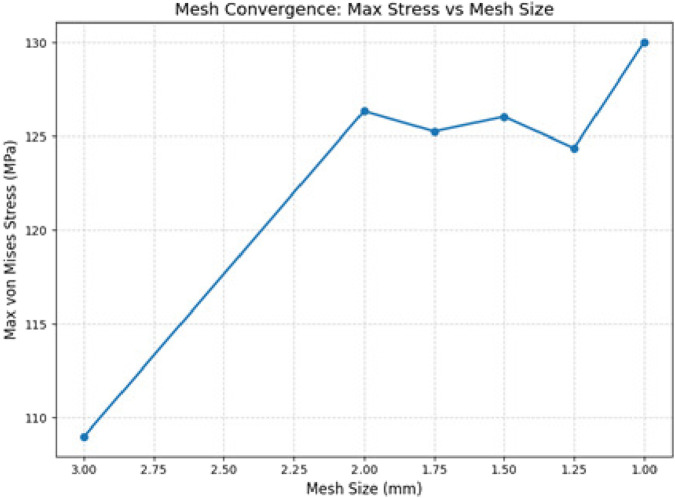
Mesh convergence study on the finite element model.

Similarly, this assembly was performed for all seven models in Fusion 360. They were exported to ANSYS Workbench for analysis, and material properties were applied, as shown in Table 1 (Ma et al., 2022; Alkhatib et al., 2019; Abd-Elaziem et al., 2024). Meshing was carried out for the intact bone (humerus) and the humerus assembled with porous and solid stems. All parts of the 3D model, the cortical bone, trabecular bone, and humeral stem, were discretized using 10-node quadratic tetrahedral elements (ANSYS SOLID187) (Figure 5). A mesh refinement study was carried out by determining the maximum von Mises stress values for each mesh level. When the mesh size reached a range between 1.75 mm and 1.25 mm, a stable response was observed with marginal changes in stress (−0.86%, +0.63%, and −1.33%), indicating that further refinement was not needed. Since the change between the 1.25-mm and 1.0-mm meshes was 4.53%, which is below the usual 5% threshold, the 1.0-mm mesh with 186,285 elements was adopted for all subsequent analyses as an accurate and conservative management. The convergence trend is shown in Figure 6. The contact between the bone–implant interface was set as bonded as the main objective was to evaluate stress shielding (Gok, 2022), (Çelik et al., 2017).

**TABLE 1:** List of material properties of bone and implants.

Material	Mechanical property	
	Young’s modulus (GPa)	Poisson ratio (ν)
Cortical bone	20	0.3
Cancellous bone	5	0.3
Ti-6Al-4V	110	0.3
316LSS	193	0.3

### 2.4 Applied loads and boundary constraints

Considering the activities that involve the shoulder, such as reaching overhead, lifting, and forward flexion, different joint angles with four loading conditions were simulated, as presented in Table 2. Bergmann et al. (2007) investigated and reported *in vivo* contact forces and moments of shoulder implants based on the weight of a single patient. Westerhoff et al. (2009) ascertained the physiological significance of the study by Bergman et al. on four individuals by comparing the force and moments, thus validating the applied loading conditions. The three-dimensional components (Force x, Force y, and Force z) and respective moments were reconstructed from the resultant force and load orientation. This combined force was applied at the center of rotation of the humeral stem, which corresponds to the anatomical location where the joint reaction force reaches the humerus from the glenoid. This method confirms that both the magnitude and application point of the loads correspond to the physiological loading condition of the shoulder joint. The forces and moments by Bergmann et al. are expressed as the percentage of body weight of a 100-kg person. The percentage of body weight was converted to Newton using a reference body weight of 80 kg, and the resultant force and magnitudes that correspond to the position of the arm were obtained. Appropriate forces and corresponding moments were applied to the bone–implant assembly. The restraint was fixed on the distal humerus, and loading was introduced to the resected portion of the humeral head, as shown in Figure 5.

**TABLE 2:** Net forces and moments for various joint angles of the shoulder.

Joint angle	Force x (N)	Force y (N)	Force z (N)	Force net (N)	Moment x (N·mm)	Moment y (N·mm)	Moment z (N·mm)	Moment net (N·mm)
45° abduction	164.8	−345.3	125.6	400.2	1730	1,490	1,100	2,350
75° abduction	266.8	−580.8	196.2	667.1	2,280	2,120	1880	2,750
90° flexion	243.3	−525.8	180.5	612.1	1,020	630	−940	780
120° flexion	400.2	−839.7	290.2	949.6	2,350	1730	2,830	4,080

### 2.5 Stress shielding analysis

**FIGURE 7:**
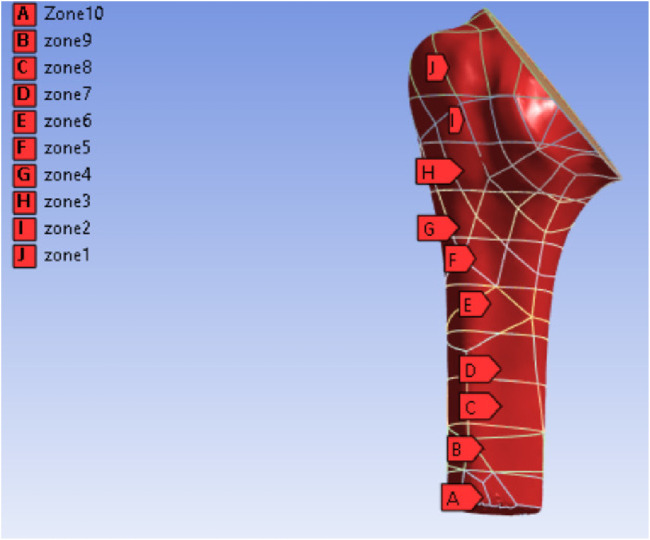
Humeral bone–implant assembly divided into ten zones.

Finite element analysis was performed on the intact humerus, assembled models (with solid and porous stem) for all the loading joint forces and moments. For each analysis, the bone model was divided into 10 zones, as shown in Figure 7; the results were computed for cortical and trabecular bones separately. The first five zones were averaged for the trabecular region, and all the 10 zones were averaged for the cortical bone. Zone-wise results of von Mises stress, volume, and strain energy were extracted. The stress shielding analysis for the study was based on the framework by Hitchon et al. (2024) applied to the reverse shoulder implants. Three parameters were assessed to calculate stress shielding and perform a comparative analysis of the biomechanical effects of various implant designs.

- Von Mises stress distribution was obtained to calculate the stress distribution in bone using ASTM F2028 (Harman et al., 2005) (method for assessing dynamic stability, loosening, or dissociation of the implant), where 750N of axial compressive load was applied to the humerus.
- The weighted mean difference of von Mises stress was determined by considering the volume of each bone element and computed between the reconstructed and native bone models (Equation 1) (Hitchon et al., 2024).

WM=∑σ×VolumeWMRecon∑σ×VolumeWMIntact×100, (1)

where WM is the weighted mean; von Mises stress (σ) is calculated at each element.

iii. Strain energy density (SED) of individual elements was calculated; this helps calculate the bone volume percentage that exhibits potential for osteointegration or osteolysis. The SED of the reconstructed bone element and intact bone element was calculated. A threshold of ±55% SED was used to determine the regions where bone can be formed or resorbed. Strain-adaptive remodeling was first proposed by Neuert and Dunning (2013), facilitating derivation of accurate predictions of bone adaptation in the distal ulna. Reeves et al. (2019) and Soltanmohammadi et al. (2022) investigated the mechanobiological responses of bone. Hence, this serves as a predictive criterion for bone remodeling (Equation 2).

$$SED=0.5\times \left(\sigma .\;\varepsilon \right),$$ (2)

where σ = stress.

Ɛ = strain.

### 2.6 Verification and validation

#### 2.6.1 Verification

A study on mesh convergence was performed to ascertain the numerical accuracy of the FEA model, as detailed in Section 2.3. The mesh was refined until the peak von Mises stress fell within the tolerance limit. A mesh size of 1 mm was selected based on this study.

#### 2.6.2 Validation

Clinical investigations related to RSA have represented stress shielding at the proximal humerus using radiographic zone classifications. In the RSA population, Nakazawa et al. (2023) applied a five-zone system, where bone resorption was evident in zone 3, which corresponds to the proximal region. For anatomic shoulder arthroplasty, Aibinder et al. (2023) adopted a six-zone scheme and identified the medial calcar and greater tuberosity as regions of osteolysis. Similarly, Denard et al. (2018) reviewed published radiographic evidence of cortical thinning and proximal bone resorption and outlined a six-zone radiographic framework for evaluating stress shielding. Despite the differences in the classification of zones, all the studies emphasize that the proximal metaphysis is the most common area for bone loss.

Our study uses 10-zone categorization, motivated by Hitchon et al. (2024) (with an eight-zone layout), to investigate a finer resolution of load distribution patterns along the humerus. The upper zones, which correspond to the proximal metaphyseal region, are found to be subjected to decreased load transfer. Within this region, the elliptical porous stem demonstrated reduced stress, similar to the patterns of proximal stress shielding, as recorded in clinical imaging investigations. Conversely, the trabecular pattern showed higher stress levels, suggesting that a near-to-natural load sharing can lower the probability of bone resorption. Circular stems showed moderate results between the two extremes. This pattern clearly aligns with the findings of Hitchon et al. (2024), demonstrating that porous stem configurations are able to sustain proximal loading than the solid stems, thereby supporting the clinical relevance of the trabecular design.

## 3 Results

### 3.1 Von Mises stress

**FIGURE 8:**
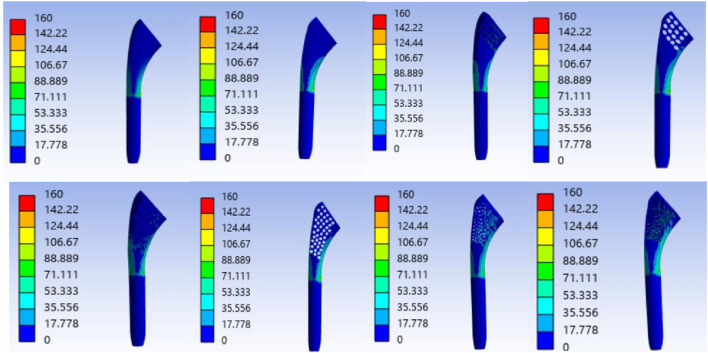
Von Mises stress at 750N solid, elliptical, circular, and trabecular stems with Ti alloy and 316LSS, respectively.

Von Mises stress distribution in all eight implant models at 750 N is shown in Figure 8. The trabecular implants (titanium and 316L SS) show lower stress values (90 MPa and 96 MPa), followed by the circular, elliptical, and solid implants. All porous titanium implants (Ti6Al4V) and all porous 316L SS implants showed yield stress below 880 MPa and below 205 MPa for Ti6Al4V and 316L SS, respectively.

### 3.2 Weighted mean difference (by volume) in von Mises stress

**FIGURE 9:**
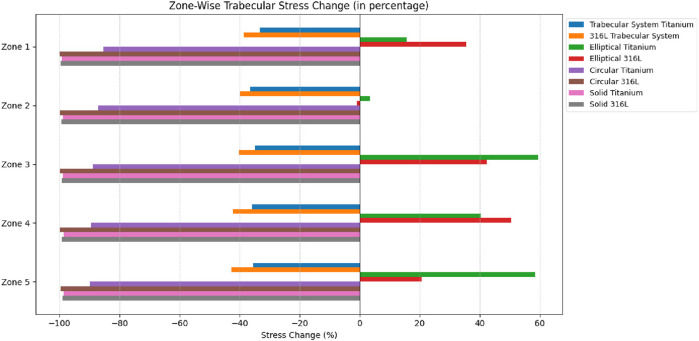
Weighted mean difference by volume for trabecular bone.

**FIGURE 10:**
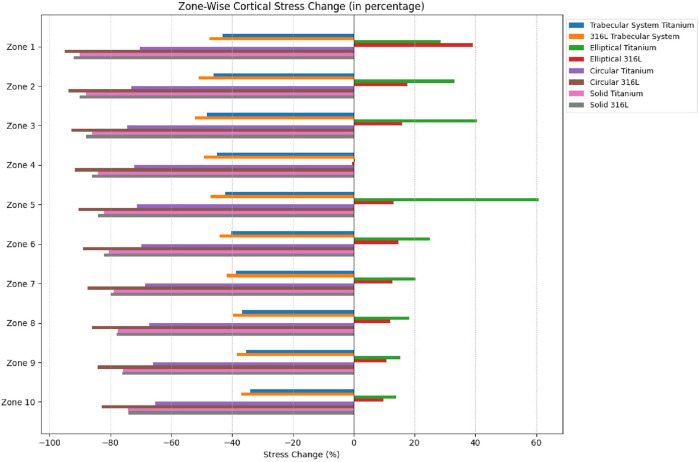
Weighted mean difference by volume for cortical bone.

Weighted mean difference by volume in von Mises stress was calculated for trabecular and cortical bones for all implant designs as a percentage of the intact stress, as shown in Figures 9, 10, respectively.

The positive changes indicate stress overloading for the models, and the negative values signify the effect of stress shielding. The titanium trabecular implant indicated −32.2% changes in trabecular stress and −41.6% in cortical stress, and the 316LSS trabecular implant exhibited – 27.6% trabecular and −55.45% cortical stress changes. On the contrary, circular titanium and circular 316LSS showed trabecular stress changes of −34.3% and −33.3%, along with cortical stress changes of −58.8% and −58.06%, respectively. Elliptical titanium and elliptical 316LSS demonstrated trabecular stress changes of −37.3% and −38.09%, respectively, while cortical stress increased by −67% and −70.7%. Solid titanium and solid 316LSS implants showed trabecular stress changes of −42% and −43%, along with cortical stress changes of −74% and −77.0%, respectively.

**FIGURE 11:**
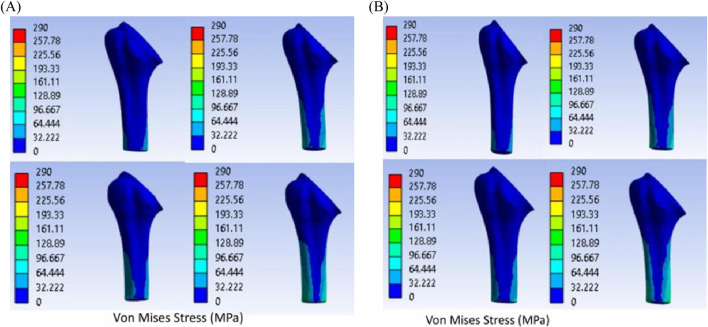
Von Mises stress: **(A)** humeral bone–titanium trabecular implant; **(B)** humeral bone–316LSS trabecular implant.

Von Mises stress of the titanium and 316L SS trabecular bone–implant assembly is presented in Figure 11. These studies indicate that the titanium trabecular system and 316LSS trabecular implant reduce the effects of stress shielding and exhibit a behavior close to the intact bone compared with other models. The FEA images use a color scale that is calibrated with warmer colors representing high values and cooler hues indicating low values.

### 3.3 Bone formation and resorption metrics

**FIGURE 12:**
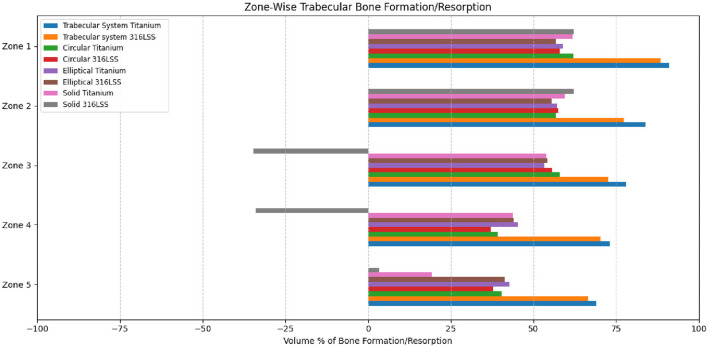
Percentage of bone formation/resorption (by volume) in trabecular bone.

**FIGURE 13:**
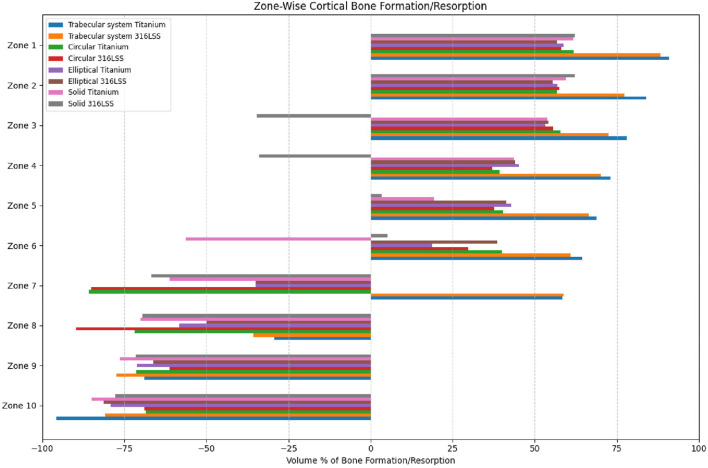
Percentage of bone formation/resorption (by volume) in cortical bone.

The volume percentage of bone formation, resorption, and unchanged status is shown in Figures 12, 13 for trabecular and cortical bones, respectively.

**FIGURE 14:**
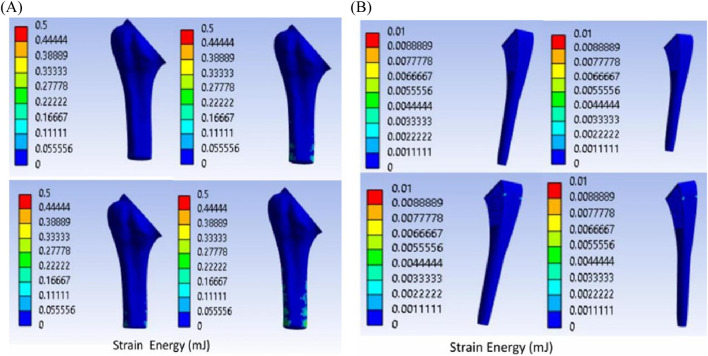
Strain energy: **(A)** humerus–trabecular titanium implant **(B)** trabecular titanium implant.

**FIGURE 15:**
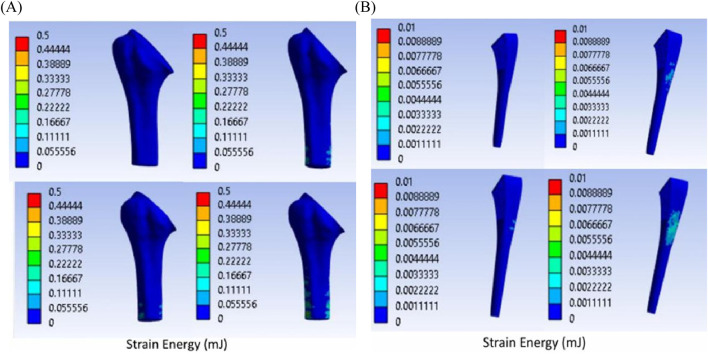
Strain energy: **(A)** humerus–trabecular 316LSS implant **(B)** trabecular–316LSS implant.

Titanium trabecular bone shows good formation across most of the zones, followed by 316LSS trabecular bone. Circular titanium undergoes bone formation in the proximal zones, and in the mid-zones, they do not undergo either bone formation or resorption, remaining unchanged. In the distal zones, they have total resorption. Similarly, circular 316LSS behaves in the same manner with few variations in bone resorption and formation; however, its performance is inferior to that of circular titanium. Proximally, elliptical titanium and elliptical 316LSS show regions of very little bone formation, and in between, many regions exhibit no changes, while the distal regions undergo resorption. In contrast, solid titanium shows bone formation in the first two zones, and the middle zone remains unchanged, while from zone 6, it undergoes complete resorption. Likewise, solid 316LSS undergoes bone formation and remains unchanged, and after zone 7, it undergoes complete resorption. The strain energy plots of humerus–trabecular titanium and humerus–trabecular 316LSS assemblies and strain energy plots of trabecular titanium and trabecular 316LSS implants are represented in Figures 14, 15, respectively.

**FIGURE 16:**
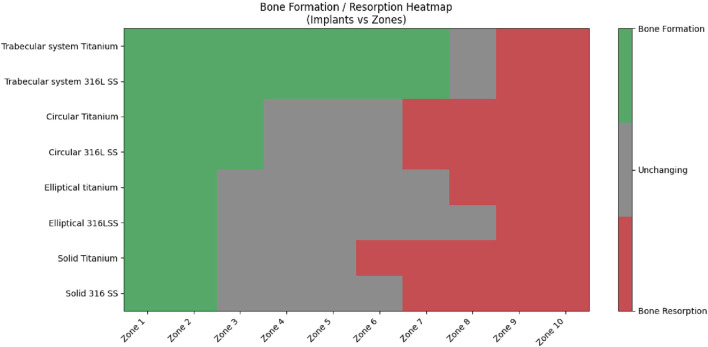
Spatial distribution of bone formation/resorption/unchanged.

Figure 16 shows the regional distribution of the potential of bone formation/resorption/unchanged for all tested implant designs. The trabecular system of titanium and 316SS were found to have superior bone formation potential in 7 of 10 zones. Conversely, circular and elliptical designs exhibited few bone formation zones, whereas solid implants contributed more to bone resorption than to bone formation. This signifies that the titanium trabecular system had a superior performance to that of other designs and reflects a favorable mechanical stimulus for osteointegration across the proximal and distal regions.

## 4 Discussion

We aimed to understand the behavior of the porous reverse shoulder implants with that of their fully solid counterparts. The present analysis indicates that the design and the materials considerably influence the von Mises stress patterns in the reverse shoulder humeral stems. Von Mises stress of 750 N N indicates that implants with a porous trabecular architecture have a better stress profile of 90 MPa compared to solid stems with a stress of 152 MPa. These implants with trabecular structures mimicking the bone exhibited better bone remodeling, especially in the upper zones of the cortical bone. This result is consistent with the findings of Hitchon et al. (2024) and Gok (2022), demonstrating that porous humeral stems transfer more load to the bone and reduce bone loss in the cortical region. Likewise, investigations by Reeves et al. (2019) and Soltanmohammadi et al. (2022) proved that the implementation of porous implants can result in improved osseointegration of the bone–implant assembly by creating a balance in load transfer to the bone. However, solid implants demonstrated high stress shielding in the cortical and trabecular regions, inducing long-term resorption. In addition, these findings were also consistent with those of Neuert and Dunning (2013) and Gok M(41), proving that high stress shielding can disrupt the process of bone remodeling, resulting in loosening of the prosthesis. The weighted mean average of von Mises stress (by volume) across studies supports that porous trabecular implants, particularly the trabecular titanium systems, retained mechanical stimulation close to that of the intact bone, compared with other implant designs. Therefore, these investigations help optimize the performance of porous implants with trabecular architecture compared to conventional designs. The outcome of the study based on strain energy density reveals that trabecular titanium had extensive bone formation zones of > +55% in both cortical and trabecular bones. Conversely, it is evident that solid stems showed large-scale bone resorption zones of < −55%, resulting in potential high stress shielding. These studies correspond to the prior works by Soltanmohammadi et al. (2022) and Hitchon et al. (2024), where hollow-stemmed humeral implants (Soltanmohammadi et al., 2022) and porous implants (Hitchon et al., 2024) have been compared to solid implants, resulting in less stress shielding.

Hitchon et al. demonstrated that uniformly distributed porous stems resulted in a loss of mechanical stimulus in the upper segment of the humerus than in the lower segment. Therefore, they suggested that implementing a porosity gradient in the proximal zone of the humerus may transfer natural load to the bone. Soltanmohammadi et al. (2022) also proved that the performance of hollow humeral stems was superior to that of solid stems in the proximal slice. These findings have been reported in parallel in femoral stems by Liu et al. (2021), where an axial gradient was able to reduce stress shielding in the proximal region of the femur compared with a prosthesis featuring a uniform porous design. Investigations in hip implants by Alkhatib et al. (2019) indicated that porous femoral stems enhance physiological load transfer and lower bone loss in the proximal femur, emphasizing that both stiffness and design architecture play a significant role in bone remodeling.

Overall, these studies prove that porosity introduced in the proximal region of RTSA humeral stems provides better clinical and biomechanical advantages through mechanical stimulation and remodelling while protecting implant longevity. A key limitation is that cortical bone was treated as an isotropic material. Although cortical bone behaves differently in various directions, numerous finite element model studies use isotropic properties because specimen-specific orthotropic data are not always available. Although this assumption might influence the exact stress values in the cortical bone, it is unlikely to affect comparative results between the different stem designs used in this study. Other limitations of the study are that implants with three varying porosities were chosen, but a porosity spectrum was not evaluated. In addition, using a non-specific bone model limits its application in patients with degenerative conditions such as arthritis and osteoporosis, which can directly contribute to changes in the stress profile and bone formation.

## 5 Clinical implications

The outcome of the study has significant implications for clinical investigations, surgeons, and patients. Porosity in humeral stems is beneficial to surgeons as they maintain better load transfer to the proximal metaphysis, which helps preserve the bone stock, thereby decreasing complications. Avoiding problems related to cortical thinning, bone resorption, and unstable fixation is vital for surgeons as they lead to technical challenges, leading to revision surgeries. Among porous configurations, trabecular lattice designs are advantageous as they support metaphyseal loading and promote better protection of proximal bone.

Porous humeral stems enable patients to maintain stronger bone around the implant, contributing to shoulder stability over time. Trabecular stem is favorable as its lattice structure enhances natural bone loading and minimizes the effect of loosening. These outcomes are particularly beneficial for younger and more active patients who place long-term mechanical demands on the implant. By preserving proximal humeral bone, porous designs can lower the likelihood of revision surgeries, offering a durable shoulder replacement.

The models used in this study provide a clinical relevance to the behavior of porous architectures on the metaphyseal load transfer in RSA. The zone-based approach to evaluate the effect of stress shielding allows early identification of designs, which help determine the long-term behavior of the humeral stem. The model’s clear differentiation between the healthier proximal loading by the trabecular design and the reduced loading associated with the elliptical pattern shows its usefulness in refining the stem architecture and helping surgeons choose designs that are more likely to preserve bone.

## 6 Conclusion

This work exemplifies that RTSA humeral stems with porosity in the proximal zones can efficiently increase the mechanical stimulus and accelerate bone remodeling in the proximal zone of the humerus. Analysis of implants with varying porosity shapes revealed that titanium stems with a trabecular architecture has a better stress profile, providing the desired outcome of porous implants. Future work should involve evaluating RSA humeral stems with different porosity gradients and varying bone densities, alongside testing fatigue and micromotion, to corroborate biological and structural integrity.

## Data Availability

The CT images analyzed in this study are publicly available on Kaggle (https://www.kaggle.com/datasets/syxlicheng/automatically-transform-ct-datasets-into-drrs). Further inquiries can be directed to the corresponding author.
